# Anemia and Systemic Inflammation Rather than Arterial Circulatory Dysfunction Predict Decompensation of Liver Cirrhosis

**DOI:** 10.3390/jcm9051263

**Published:** 2020-04-26

**Authors:** Christina Bothou, Sabrina Rüschenbaum, Alica Kubesch, Leonie Quenstedt, Katharina Schwarzkopf, Christoph Welsch, Stefan Zeuzem, Tania Mara Welzel, Christian Markus Lange

**Affiliations:** 1 Department of Internal Medicine 1, Goethe-University Hospital Frankfurt, Theodor-Stern-Kai 7, 60590 Frankfurt, Germany; sabrina.rueschenbaum@uk-essen.de (S.R.); alica.kubesch@kgu.de (A.K.); leonie@quenstedt-hamburg.de (L.Q.); Katharina.Schwarzkopf@kgu.de (K.S.); christoph.welsch@kgu.de (C.W.); stefan.zeuzem@kgu.de (S.Z.); tmwelzel@web.de (T.M.W.); christian.lange@uk-essen.de (C.M.L.); 2 Klinik für Endokrinologie, Diabetologie und Klinische Ernährung, UniversitätsSpital Zürich, Forschungslabor, Wagistrasse 21, 4. OG, CH-8952 Schlieren, Switzerland

**Keywords:** portal hypertension, ascites, acute-on-chronic liver failure, decompensated liver cirrhosis

## Abstract

Background: While systemic inflammation is recognized as playing a central role in the pathogenesis of organ failures in patients with liver cirrhosis, less is known about its relevance in the development of classical hepatic decompensation. Aim: To characterize the relationship between systemic inflammation, hemodynamics, and anemia with decompensation of liver cirrhosis. Methods: This is a post-hoc analysis of a cohort study of outpatients with advanced liver fibrosis or cirrhosis. Results: Analysis included 338 patients of whom 51 patients (15%) were hospitalized due to decompensation of liver cirrhosis during a median follow-up time of six months. In univariate analysis, active alcoholism (*p* = 0.002), model of end-stage liver disease (MELD) score (*p* = 0.00002), serum IL-6 concentration (*p* = 0.006), heart rate (*p* = 0.03), low arterial blood pressure (*p* < 0.05), maximal portal venous flow (*p* = 0.008), and low hemoglobin concentration (*p* < 0.00001) were associated with hospitalization during follow-up. Multivariate analysis revealed an independent association of low hemoglobin (OR = 0.62, 95% CI = 0.51–0.78, *p* = 0.001) and serum IL-6 concentration (OR = 1.02, 95% CI = 1.01–1.04, *p* = 0.03)—but not of hemodynamic parameters—with hepatic decompensation. An inverse correlation between hemoglobin concentration and portal venous flow (*R* = −0.362, *p* < 0.0001) was detected for the non-hospitalized patients. Accuracy of baseline hemoglobin levels for predicting hospitalization (AUC = 0.84, *p* < 0.000001) was high. Conclusion: Anemia and systemic inflammation, rather than arterial circulatory dysfunction, are strong and independent predictors of hepatic decompensation in outpatients with liver cirrhosis.

## 1. Introduction

Liver cirrhosis is characterized by high mortality and constitutes the second leading cause of digestive track disease-related deaths [1]. According to the World Health Organization, liver cirrhosis is responsible for about 170,000 deaths per year in Europe [2]. Liver cirrhosis is also associated with significant morbidity and leads to more than 150,000 hospital admissions and costs the state $4 billion per year [3]. In the natural course of the disease, liver cirrhosis is characterized by a compensated stage followed by a decompensated stage, through the development of one of the following complications: variceal hemorrhage, ascites, encephalopathy, or jaundice. The transition from compensation to decompensation dramatically affects the median survival time of the patients, from over 12 years to less than 2 years, respectively [4,5,6]. Consequently, efforts to prevent the progression from compensated to decompensated cirrhosis and to understand the underlying mechanisms are highly important [7].

Portal hypertension, peripheral vasodilation, and liver insufficiency are considered key elements in the pathogenesis of decompensation of liver cirrhosis [8]. Portal hypertension and cirrhosis-associated intestinal dysbiosis result in intestinal translocation of bacteria and bacterial products to the portal venous blood and extraintestinal organs, where they contribute to a cirrhosis-associated chronic inflammatory state [9]. Furthermore, patients with liver cirrhosis are intrinsically at increased risk of infection and sepsis, which can further fuel systemic inflammation [9]. The role of systemic inflammation is increasingly recognized as an important determinant of the development of complications of liver cirrhosis [10]. In this regard, it is particularly well established that systemic inflammation and circulatory failure are hallmarks and interconnected in the development of acute-on-chronic liver failure (ACLF) [11]. In contrast, a better understanding of the contribution of systemic inflammation and circulatory dysfunction to disease progression and hepatic decompensation in outpatients with relatively stable liver cirrhosis is still needed.

Anemia is another factor which has recently been described to predict adverse outcomes in patients with liver cirrhosis, namely the development of ACLF in outpatients with liver cirrhosis, as well as mortality of patients with hepatocellular carcinoma [12,13]. In a previous analysis of this cohort study, which was dedicated to exploring the relationship between vitamin D deficiency and hepatic decompensation during follow-up, baseline hemoglobin was independently associated with hepatic decompensation [14]. Yet, the relationship between anemia, systemic inflammation, and hemodynamics in patients with liver cirrhosis has not been systematically assessed.

In view of these findings, we aimed to assess the role of systemic inflammation, anemia, and systemic and portal venous hemodynamics in the prediction of hepatic decompensation in a large cohort study of outpatients with advanced liver fibrosis and cirrhosis.

## 2. Patients and Methods

### 2.1. Study Population

Since July 2016, all consecutive patients presenting to the outpatient liver clinic of the University Hospital Frankfurt, Germany, with advanced liver fibrosis or cirrhosis were offered to participate in this prospective cohort study, as described previously (Kubesch et al.) [14]. Inclusion criteria were age older than 18, the presence of liver fibrosis grade ≥F2 (Metavir) or cirrhosis and written informed consent to participate in the study. Patients were excluded if they were younger than 18 years old, pregnant, or breastfeeding, if hepatocellular carcinoma (HCC) beyond Milan was diagnosed, if they were infected with human immunodeficiency virus, or in the case of therapy with immunosuppressive agents. In addition, patients with end-stage cardiovascular disease, severely impaired renal function not due to hepatorenal syndrome, other cancer than HCC, or severe neurologic disease were excluded. Patients were followed up every three months and when admitted to hospital. On each follow-up visit, clinical and laboratory variables were obtained and serum samples were stored for further analyses.

All patients from this cohort study were included in the present post-hoc analyses if they were recruited until August 2017 and followed until November 2017 and if sufficient serum baseline samples for IL-6-quantification were available.

Liver fibrosis was assessed by shear-wave elastography and FibroTest^TM^, as described (Kubesch et al.) [14]. Acute decompensation of liver cirrhosis was defined as presence of one of the following criteria: hepatic encephalopathy (HE) of grade I to IV according to West Haven Criteria [15], portal hypertensive bleeding diagnosed by emergency endoscopy [16], spontaneous bacterial peritonitis (SBP) or other bacterial infections requiring hospitalization (all these patients had ascites or variceal bleeding) [17], clinically evident ascites documented on physical examination as moderate (grade 2) or large ascites (grade 3), according to the classification of the International Ascites Club, with or without acute kidney injury (AKI), defined by a serum creatinine increase of over 0.3 mg/dL or by 50% over baseline [18].

### 2.2. Hemodynamic Assessment

Heart rate as well as systolic and diastolic blood pressure (assessed by Riva-Rocci method) were assessed at baseline in resting conditions [19]. Mean arterial blood pressure was calculated using the following formula: (2 × diastolic blood pressure + systolic blood pressure)/3. Maximal portal venous flow was quantified by Doppler sonography [19,20].

### 2.3. Quantification of IL-6 Serum Levels

Serum concentrations of IL-6 were quantified by ELISA (R&D Systems, Minneapolis, MN, USA). Of note, the relationship between serum IL-6 concentrations and vitamin D serum levels of a subgroup of 94 patients of this cohort study were published previously [14].

### 2.4. Statistical Analyses

Statistical analyses were performed using BiAS, Version 11.06 (epsilon-Verlag, Frankfurt, Germany), and GraphPad PRISM5 (GraphPad Software, Inc., San Diego, CA, USA). Group differences were assessed by means of χ^2^ contingency tables or Wilcoxon–Mann–Whitney U tests, as appropriate. *p* values < 0.05 were considered to be statistically significant. Associations of outcomes with continuous or dichotomic variables were assessed in linear and logistic regression models, respectively. After univariate analyses, multivariate analyses were performed for significant associations using a *p* value > 0.15 for removal from the model.

## 3. Results

### 3.1. Patient Characteristics

In total, 338 patients with advanced liver fibrosis or cirrhosis were included according to the above described inclusion criteria. Baseline characteristics of these patients are depicted in Table 1. Out of the 338 patients who were included in our study, 51 patients (15%) developed decompensation of liver cirrhosis during a median follow-up time of six months (range 3–12 months) and were hospitalized for this reason. In detail, hepatic decompensation events included 23 episodes of acute and large-volume ascites, 16 episodes of significant hepatic encephalopathy, 7 infections (in combination with ascites or variceal bleeding), 16 cases of renal failure (in combination with ascites), and 6 cases of upper GI bleeding. Of the 51 subjects of this subcategory, 15 died in hospital as a consequence of hepatic decompensation. Furthermore, 36 and 42 patients who were hospitalized due to hepatic decompensation had a previous history of ascites and overall hepatic decompensation, respectively, whereas 105 and 130 patients without hospitalization at follow-up during to the study period had a previous history of ascites and overall hepatic decompensation, respectively.

### 3.2. Anemia, but Not Markers of Systemic Inflammation, Correlates with Baseline Hemodynamics

**Figure 1:**
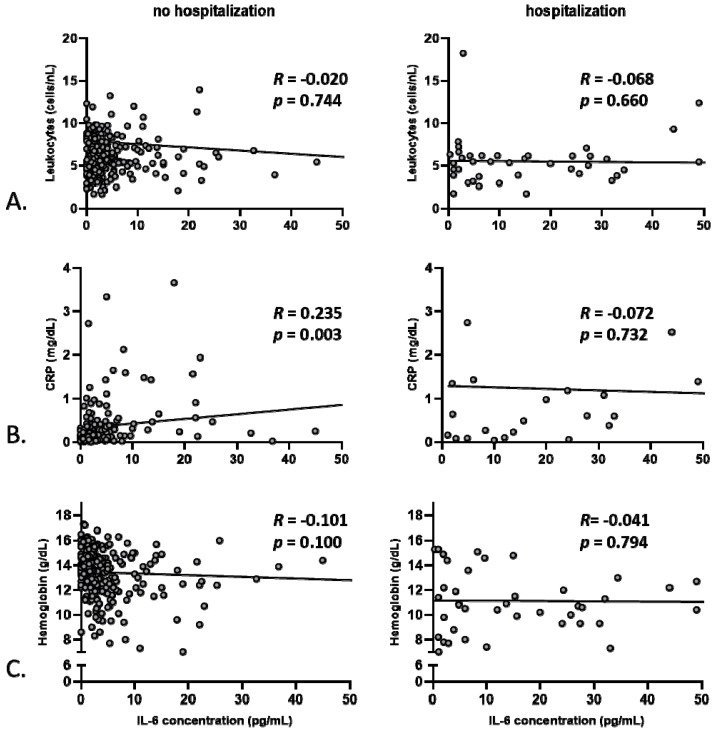
Correlation between IL-6 concentration with other markers of systemic inflammation as well as with hemoglobin levels for the hospitalized and non-hospitalized group of patients. (**A**) Correlation between baseline IL-6 concentration and leukocyte counts, CRP concentration (**B**) as well as with hemoglobin concentration (**C**).

**Figure 2:**
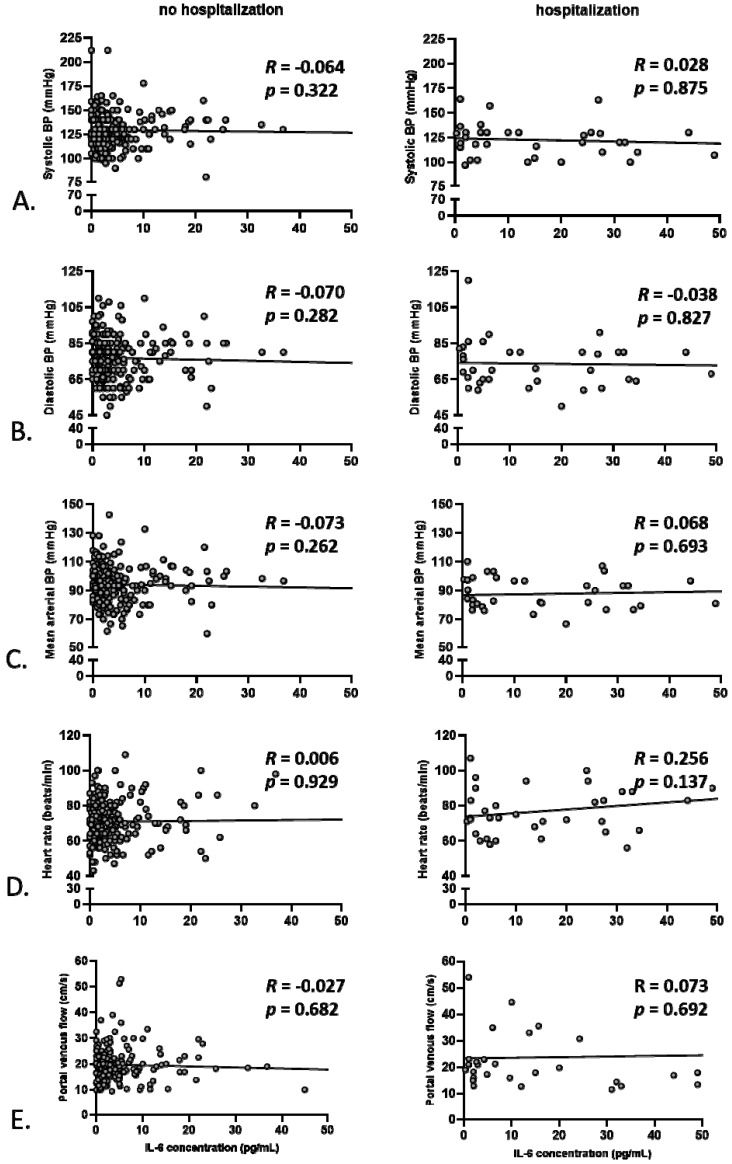
Correlation between IL-6 concentration and parameters of systemic and portal venous hemodynamics for the hospitalized and non-hospitalized group of patients. Correlations are shown between baseline IL-6 concentration and systolic (**A**), diastolic (**B**), and mean arterial blood pressure (**C**), as well as heart rate (**D**), to assess systemic circulation, as well as with maximal portal venous flow (**E**), as a surrogate parameter of portal hypertension.

**Figure 3:**
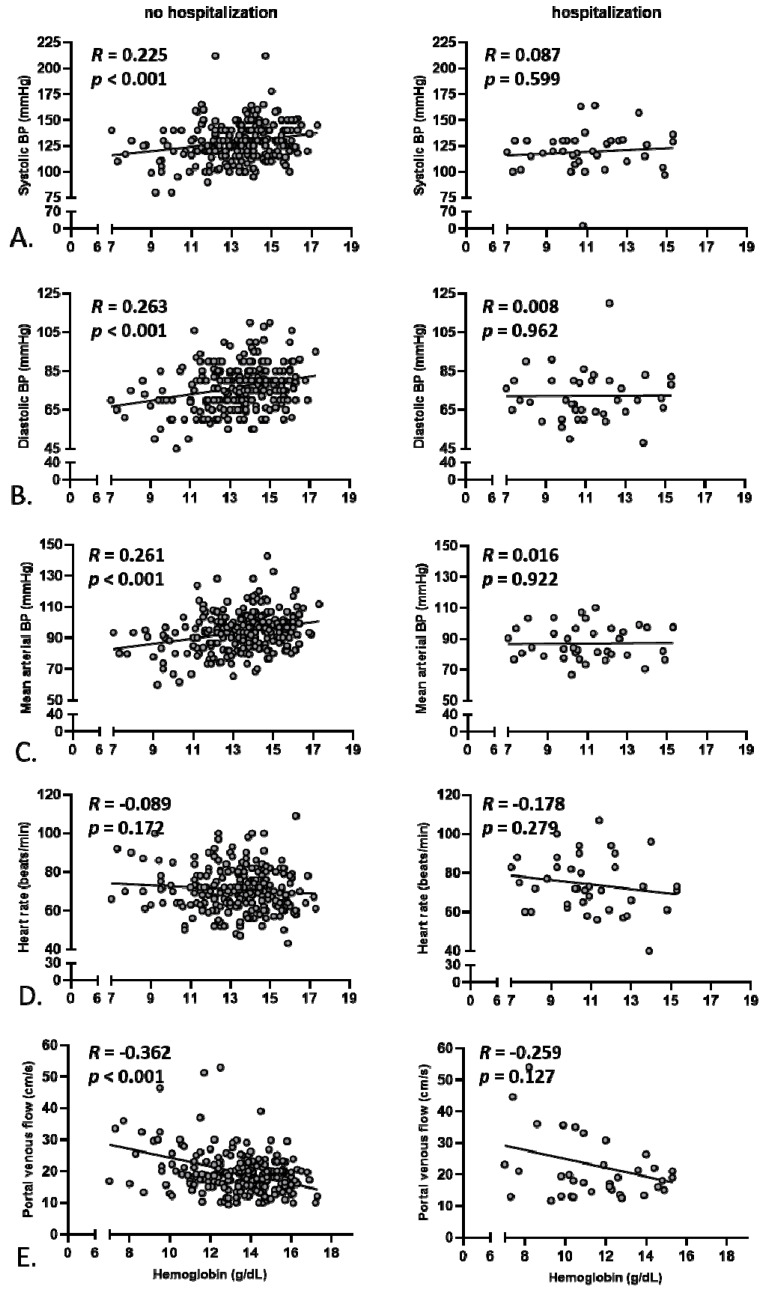
Correlation between hemoglobin concentration and parameters of systemic and portal venous hemodynamics for the hospitalized and non-hospitalized group of patients. Correlations are shown between baseline hemoglobin concentration and systolic (**A**), diastolic (**B**), and mean arterial blood pressure (**C**), as well as heart rate (**D**), to assess systemic circulation, as well as with maximal portal venous flow (**E**), as a surrogate parameter of portal hypertension.

To better explore the relationship between systemic inflammation, portal venous/systemic hemodynamic parameters, and anemia, Pearson’s correlations were performed based on baseline values for the two subgroups of patients, namely hospitalized and non-hospitalized for decompensation during the study. Of note, there was a good correlation between IL-6 and CRP serum concentrations only for the non-hospitalized patients *(R* = 0.235, *p* = 0.003 vs. *R* = −0.072, *p* = 0.732, Figure 1B), whereas no significant correlation between baseline IL-6 and blood leukocytes was observed for both groups (Figure 1A). Given the described power of serum IL-6 levels to predict mortality of patients with liver cirrhosis [21], this parameter was chosen to further assess the level of systemic inflammation in our study. No correlation between baseline IL-6 and hemoglobin levels was detected (Figure 1C). Similarly, no significant correlations between IL-6 serum levels and systolic blood pressure, diastolic blood pressure, mean arterial blood pressure, heart rate, or portal venous flow were observed for any of the subgroups (Figure 2). These observations hold true for subgroup analyses of patients with or without concomitant beta-blocker therapy for the prevention of variceal bleeding (correlation between IL-6 and mean arterial pressure, without beta-blocker: *R* = −0.05, *p* = 0.5, with betablocker: *R* = −0.001, *p* = 0.9; heart rate, without beta-blocker: *R* = 0.07, *p* = 0.3, with betablocker: *R* = −0.1, *p* = 0.6; portal venous flow, without beta-blocker: *R* = −0.05, *p* = 0.5, with betablocker: *R* = −0.2; *p* = 0.4). However, for the non-hospitalized patients, a robust positive correlation was observed between blood hemoglobin levels and systolic as well as diastolic blood pressure, and hemoglobin levels correlated inversely with portal venous flow (Figure 3). For the hospitalized subgroup, these correlations were not observed. Additionally, for the whole group, an inverse correlation between spleen size and hemoglobin was detected (*R* = −0.18, *p* = 0.002).

### 3.3. Predictors of Hospitalization Due to Hepatic Decompensation

The above described analyses suggest an association between anemia and hemodynamic parameters in outpatients with liver cirrhosis, whereas no relevant association between systemic inflammation and hemodynamics was observed. To further explore these relationships and to assess their impact on the risk of subsequent decompensation of liver cirrhosis, uni- and multivariate regression analyses were performed to identify baseline predictors of the risk of hospitalization due to hepatic decompensation during follow-up. In univariate analysis of variables at baseline, active alcoholism (*p* = 0.002), model of end-stage liver disease (MELD) score (*p* = 0.00002), serum IL-6 concentration (*p* = 0.006), heart rate (*p* = 0.03), and maximal portal venous flow (*p* = 0.008) were positively associated with hepatic decompensation, whereas systolic blood pressure (*p* = 0.02), diastolic blood pressure (*p* = 0.04), and hemoglobin concentration (*p* < 0.00001) were inversely associated with the risk of hepatic decompensation during follow-up (Table 2). In this regard, the strongest associations with respect to the odds ratios were observed in the presence of alcoholic liver disease (OR = 2.70, 95% CI = 1.41–5.15), hemoglobin (OR = 0.57, 95% CI = 0.47–0.70), and IL-6 concentration (OR = 2.44, 95% CI = 1.22–4.76). In multivariate analysis, active alcoholism (OR = 2.17, 95% CI = 1.00–4.77, *p* = 0.05), MELD score (OR = 1.11, 95% CI = 1.01–1.22, *p* = 0.02), hemoglobin concentration (OR = 0.62, 95% CI = 0.51–0.78, *p* = 0.001), and serum IL-6 concentration (OR = 1.02, 95% CI = 1.01–1.04, *p* = 0.03), all at baseline, were independently associated with the risk of hospitalization due to hepatic decompensation during follow-up. Of note, the association between hemoglobin concentration, IL-6 concentration, hospitalization due to hepatic decompensation, as well as the MELD score, remained significant if hepatic decompensation at baseline was included in the multivariate analysis (Model 2, Table 2).

### 3.4. Diagnostic Capacity of the Predictors

**Figure 4:**
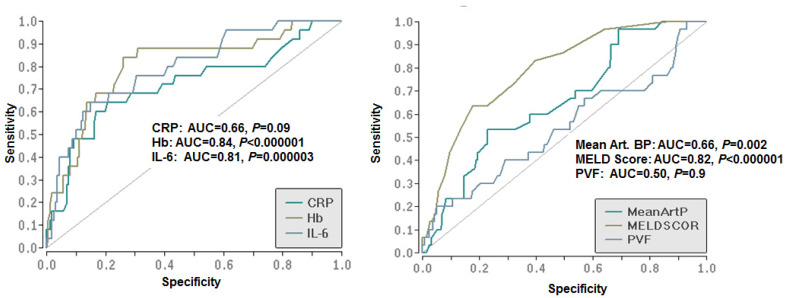
Area under the receiver operating characteristic curve (AUROC) of baseline variables predicting the risk of hospitalization due to hepatic decompensation during follow-up. CRP, C-reactive protein; Hb, hemoglobin; IL-6, interleukin-6; art. BP, arterial blood pressure; PVF, maximal portal venous flow.

Finally, receiver operating characteristic (ROC) analyses were performed to assess the capacity to predict hospitalization due to hepatic decompensation. As shown in Figure 4, hemoglobin concentration had the highest accuracy of predicting hospitalization due to hepatic decompensation (area under the curve (AUC) = 0.84, *p* < 0.000001), followed by MELD score (AUC = 0.82, *p* < 0.000001) and serum IL-6 concentration (AUC = 0.81, *p* = 0.000003). A substantially lower accuracy was observed for serum CRP levels (AUC = 0.66, *p* = 0.09) and mean arterial blood pressure (AUC = 0.66, *p* = 0.002) (Figure 4).

## 4. Discussion

The major finding of our study is that both systemic inflammation and anemia are strong and independent predictors of hospitalization due to hepatic decompensation in outpatients with liver cirrhosis, whereas markers of arterial circulatory dysfunction may play a minor role in this scenario.

Liver cirrhosis is a disease characterized by low levels of systemic inflammation already in the compensated stage, which augments during decompensation and severely exacerbates during the development of ACLF [22]. In this regard, systemic inflammation is currently considered as a major driver of ACLF-defining organ failure, as systemic inflammation activates coagulation and promotes endothelial dysfunction, tissue hypoxia, and cell death [22,23]. In contrast, the role of systemic inflammation in the pathogenesis of pure hepatic decompensation not complicated by organ failure is less clear [10]. Although our study cannot provide proof of causal relationships, the strong association between serum IL-6 levels and decompensation during follow-up might be considered as a hint of a pathophysiological role of inflammation in decompensation of liver cirrhosis.

It is an important finding that parameters reflecting arterial circulatory dysfunction at baseline, i.e., systolic and diastolic blood pressure and heart rate, were less powerful predictors of hepatic decompensation during follow-up compared to systemic inflammation. This is remarkable because arterial vasodilation is traditionally considered to be a hallmark of hepatic decompensation [10]. Furthermore, in our study, no correlation between serum IL-6 levels and parameters of circulatory dysfunction was observed. Altogether, these findings support a concept of a key role of systemic inflammation rather than arterial circulatory dysfunction in the development of decompensation of liver cirrhosis as early as prior to the progression to organ failure. In this regard, our study confirms and expands on the previously published finding that serum IL-6 levels are strongly predictive of mortality on the wait list for liver transplantation [21]. Yet, it is important to note that our study does not address the important role of portal hypertension during the development of hepatic decompensation [8], given the imperfect modalities of assessing portal hypertension in our study.

Although excessive IL-6 serum levels have been described in patients with ACLF [11], peripheral blood leukocytes have been implemented in clinical scores as markers of systemic inflammation to predict mortality of ACLF [22]. In this regard, the poor correlation between IL-6 serum levels and peripheral blood leukocytes for the patients that were hospitalized, as well as the CRP levels that we observed in the present study, is noteworthy. Only for the non- hospitalized patients could a positive correlation between CRP and IL-6 be observed and was expected from the literature. Why the same correlation was not detected in the hospitalized patients is not clear. This could be, however, explained either by a plateau in CRP or IL-6 or by the small sample number of this subgroup. At least in our study, IL-6 serum levels predicted hepatic decompensation (which we acknowledge to be a different endpoint than ACLF) better than leukocytes and CRP. IL-6 serum concentration is a well-established marker of inflammation in critically ill patients, a setting in which it may be superior to leukocytes and CRP [24]. The accuracy observed herein of serum IL-6 concentration to predict hepatic decompensation in individuals with relatively low levels of inflammation should be considered in future studies.

Although the role of anemia as a complication of liver cirrhosis is well recognized, the power of baseline anemia is predicting hospitalization due to hepatic decompensation observed in our study is remarkable. In particular, the association between baseline anemia and hepatic decompensation during follow-up was as high as between baseline anemia and systemic inflammation, and significantly higher than the MELD score or parameters reflecting arterial circulatory dysfunction. The importance of anemia in predicting adverse outcomes in patients with liver cirrhosis is in line with previous studies, showing, for example, that anemia is predictive of mortality in patients with HCC, as well as of the development of ACLF in outpatients with liver cirrhosis [12,13]. Furthermore, we observed a robust correlation between anemia and hemodynamic parameters. In fact, there may be a reciprocal relationship between anemia and circulatory dysfunction in patients with liver cirrhosis. On the one hand, portal hypertension may contribute to the pathogenesis of anemia by promoting intestinal blood loss and—possibly—malabsorption of iron, folic acid, and vitamin B12 [25,26]. In this regard, the inverse correlation between hemoglobin and spleen can be explained. On the other hand, it appears plausible that anemia contributes to arterial hypotension and tachycardia in patients with liver cirrhosis. Taking everything into account, a possible causal relationship between anemia, circulatory dysfunction, and hepatic decompensation should be investigated in future studies.

Our study has several limitations. Most importantly, we assessed only imperfect surrogate markers of portal hypertension such as maximal portal venous flow or platelet counts. Therefore, our study is not suitable for a definite assessment of the relationship between portal hypertension, systemic inflammation, and anemia. Furthermore, our study collectively is relatively heterogeneous, including patients with a broad spectrum ranging from advanced liver fibrosis to compensated and decompensated liver cirrhosis. Yet, the large number of patients included appeared to allow for a robust statistical analysis, which is also reflected by the confirmation of known predictors of decompensation such as the MELD score. In addition, some of the observed correlations were relatively weak and larger studies may be required to fully characterize the relationship between hemodynamics, anemia, and systemic inflammation. Finally, the use of rifaximin was not systematically assessed in our study, which may constitute a relevant bias in the analysis of systemic inflammation.

## 5. Conclusions

Our study suggests an important role played by systemic inflammation in the pathogenesis of decompensation of liver cirrhosis as early as before the onset of organ failures. Furthermore, our study highlights the potential of anemia (neglected until now)—and perhaps a causal relationship—in predicting hepatic decompensation.

## Figures and Tables

**Table 1:** Baseline characteristics of included patients.

	All Patients (*N* = 338)	No Hospitalization Due to Decompensation at Follow-Up (*N* = 287)	Hospitalization at Follow-Up Due to Decompensation (*N* = 51)	*p*-Value
Age (years), mean ± SD	59.7 ± 11.9	59.4 ± 12.1	61.5 ± 10.9	0.3
Male gender, *N* (%)	194 (57)	163 (57)	31 (60)	0.7
BMI (kg/m^2^), mean ± SD	27 ± 5	27 ± 5	26 ± 6	0.1
Death during follow-up, *N* (%)	16 (5)	1 (0.3)	15 (30)	<0.0001
Underlying liver disease				
NASH	33 (10)	26 (9)	7 (14)	0.4
Alcohol	103 (30)	88 (31)	15 (29)	0.9
HCV	117 (35)	102 (36)	15 (29)	0.5
Other or unclear	85 (25)	71 (25)	14 (27)	0.7
MELD score, mean ± SD	10.2 ± 4.2	9.7 ± 3.8	13.8 ± 4.4	<0.0001
Decompensation at baseline, *N* (%)	131 (39)	105 (37)	26 (51)	0.05
Prior decompensation, *N* (%)	172 (51)	130 (45)	42 (82)	<0.0001
CRP (mg/L), mean ± SD	0.53 ± 0.89	0.4 ± 0.6	1.4 ± 1.6	<0.0001
Leukocytes/nL, mean ± SD	5.98 ± 2.3	6.0 ± 2.2	5.7 ± 2.7	0.1
Platelets/nL, mean ± SD	138 ± 72	141 ± 71	123 ± 79	0.02
Hemoglobin (mg/dL), mean ± SD	13.08 ± 2.08	13.4 ± 1.9	11.2 ± 2.3	<0.0001
Creatinine (mg/dL), mean ± SD	0.9 ± 0.43	0.9 ± 0.3	1.1 ± 0.8	0.02
Albumin (g/dL), mean ± SD	3.9 ± 0.7	4.1 ± 0.6	3.3 ± 0.6	<0.0001
Bilirubin (mg/dL), mean ± SD	1.47 ± 1.52	1.3 ± 1.5	2.3 ± 1.7	<0.0001
INR, mean ± SD	1.22 ± 0.34	1.2 ± 0.3	1.4 ± 0.3	<0.0001
IL-6 (pg/mL), mean ± SD	7.8 ± 14	7.3 ± 16	24.9 ± 24	<0.0001
Systolic BP (mmHg), mean ± SD	134 ± 44	129 ± 18	119 ± 23	0.004
Diastolic BP (mmHg), mean ± SD	80 ± 49	77 ± 11	72 ± 13	0.02
Heart rate (beats/min), mean ± SD	71 ± 11	71 ± 11	75 ± 15	0.09
Max. Portal Venous Flow (cm/s), mean ± SD	19.9 ± 8.5	20 ± 7.5	23 ± 13	0.4

SD: Standard Deviation, NASH: Nonalcoholic steatohepatitis, HCV: Hepatitis C Virus, MELD: model of end-stage liver disease, CRP: C-reactive protein, BMI: body mass index, BP: blood pressure, MAP: mean arterial pressure, IL-6: Interleukin 6, INR: international normalized ratio.

**Table 2:** Logistic regression analyses of hospitalization (patients *n* = 51) due to hepatic decompensation during follow-up.

	Univariate Analysis		Multivariate Analysis			
			Model 1		Model 2 *	
	*p*-Value	OR (95% CI)	*p*-Value	OR (95% CI)	*p*-Value	OR (95% CI)
Male gender	0.6	1.18 (0.63–2.24)				
Age	0.3	1.01 (0.98–1.04)				
Active alcoholism	0.002	2.70 (1.41–5.15)	0.05	2.17 (1.00–4.77)		
Hepatic decompensation at baseline	<0.00001	9.87 (4.34–22.3)			0.01	3.4 (1.31–8.84)
Mean arterial blood pressure (mmHg)	0.008	0.96 (0.93–0.99)				
Heart rate (beats/min)	0.03	1.03 (1.00–1.07)				
Max. Portal Venous Flow (m/s)	0.008	1.87 (1.18–2.97)				
MELD score	0.00002	1.19 (1.09–1.29)	0.02	1.11 (1.01–1.22)	0.02	1.12 (1.02–1.23)
Platelets cells/nL	0.15	0.99 (0.98–1.00)				
Hemoglobin (g/dL)	<0.00001	0.57 (0.47–0.70)	0.0001	0.62 (0.51–0.78)	0.0005	0.68 (0.55–0.84)
IL-6 (pg/mL)	0.006	2.44 (1.22–4.76)	0.03	1.02 (1.01–1.04)	0.03	1.02 (1.01–1.04)

MELD, model of end-stage liver disease; IL-6, interleukin-6. * Hepatic decompensation at baseline was included in Model 2.

## References

[1] Everhart J.E., Ruhl C.E. (2009). Burden of Digestive Diseases in the United States Part III: Liver, Biliary Tract, and Pancreas. Gastroenterology.

[2] Blachier M., Leleu H., Peck-Radosavljevic M., Valla D., Roudot-Thoraval F. (2013). The burden of liver disease in Europe: A review of available epidemiological data. J. Hepatol..

[3] Talwalkar J.A. (2006). Prophylaxis with beta blockers as a performance measure of quality health care in cirrhosis. Gastroenterology.

[4] D’Amico G., Garcia-Tsao G., Pagliaro L. (2006). Natural history and prognostic indicators of survival in cirrhosis: A systematic review of 118 studies. J. Hepatol..

[5] Garcia-Tsao G., Friedman S.L., Iredale J., Pinzani M. (2010). Now there are many (stages) where before there was one: In search of a pathophysiological classification of cirrhosis. Hepatology.

[6] Ginès P., Quintero E., Arroyo V., Teres J., Bruguera M., Rimola A., Caballería J., Rodés J., Rozman C. (1987). Compensated cirrhosis: Natural history and prognostic factors. Hepatology.

[7] Garcia-Tsao G., Bosch J., Groszmann R.J. (2008). Portal hypertension and variceal bleeding-Unresolved issues. Summary of an American Association for the study of liver diseases and European Association for the study of the liver single-topic conference. Hepatology.

[8] Ripoll C., Groszmann R., Garcia–Tsao G., Grace N., Burroughs A., Planas R., Escorsell À., García-Pagán J.C., Makuch R., Patch D. (2007). Hepatic Venous Pressure Gradient Predicts Clinical Decompensation in Patients With Compensated Cirrhosis. Gastroenterology.

[9] Albillos A., Lario M., Álvarez-Mon M. (2014). Cirrhosis-associated immune dysfunction: Distinctive features and clinical relevance. J. Hepatol..

[10] Bernardi M., Moreau R., Angeli P., Schnabl B., Arroyo V. (2015). Mechanisms of decompensation and organ failure in cirrhosis: From peripheral arterial vasodilation to systemic inflammation hypothesis. J. Hepatol..

[11] Clària J., Stauber R.E., Coenraad M.J., Moreau R., Jalan R., Pavesi M., Amorós À., Titos E., Alcaraz-Quiles J., Oettl K. (2016). Systemic inflammation in decompensated cirrhosis: Characterization and role in acute-on-chronic liver failure. Hepatology.

[12] Finkelmeier F., Bettinger D., Koberle V., Schultheiss M., Zeuzem S., Kronenberger B., Piiper A., Waidmann O. (2014). Single measurement of hemoglobin predicts outcome of HCC patients. Med. Oncol..

[13] Piano S., Tonon M., Vettore E., Stanco M., Pilutti C., Romano A., Mareso S., Gambino C., Brocca A., Sticca A. (2017). Incidence, predictors and outcomes of acute-on-chronic liver failure in outpatients with cirrhosis. J. Hepatol..

[14] Kubesch A., Quenstedt L., Saleh M., Rüschenbaum S., Schwarzkopf K., Martinez Y., Welsch C., Zeuzem S., Welzel T.M., Lange C.M. (2018). Vitamin D deficiency is associated with hepatic decompensation and inflammation in patients with liver cirrhosis: A prospective cohort study. PLoS ONE.

[15] American Association for the Study of Liver Diseases and European Association for the Study of the Liver (2014). Hepatic Encephalopathy in Chronic Liver Disease: 2014 Practice Guideline by the European Association for the Study of the Liver and the American Association for the Study of Liver Diseases. J. Hepatol..

[16] Garcia-Tsao G., Abraldes J.G., Berzigotti A., Bosch J. (2016). Portal hypertensive bleeding in cirrhosis: Risk stratification, diagnosis, and management: 2016 practice guidance by the American Association for the study of liver diseases. Hepatology.

[17] Angeli P., Bernardi M., Villanueva C., Francoz C., Mookerjee R., Trebicka J., Krag A., Laleman W., Ginès P. (2018). European Association for the Study of the Liver EASL Clinical Practice Guidelines for the management of patients with decompensated cirrhosis. J. Hepatol..

[18] Angeli P., Ginès P., Wong F., Bernardi M., Boyer T., Gerbes A., Moreau R., Jalan R., Sarin S.K., Piano S. (2015). Diagnosis and management of acute kidney injury in patients with cirrhosis: Revised consensus recommendations of the International Club of Ascites. J. Hepatol..

[19] Iyriboz Y. (1995). Indirect Blood-Pressure Measurement Using the Riva-Rocci-Korotkoff Method—Reply. J. Clin. Monit..

[20] Lin L.-W., Duan X.-J., Wang X.-Y., Xue E.-S., He Y.-M., Gao S.-D., Yu L.-Y. (2008). Color Doppler velocity profile and contrast-enhanced ultrasonography in assessment of liver cirrhosis. Hepatobiliary Pancreat. Dis. Int..

[21] Remmler J., Schneider C., Treuner-Kaueroff T., Bartels M., Seehofer D., Scholz M., Berg T., Kaiser T. (2018). Increased Level of Interleukin 6 Associates with Increased 90-Day and 1-Year Mortality in Patients with End-Stage Liver Disease. Clin. Gastroenterol. Hepatol..

[22] Hernaez R., Solà E., Moreau R., Ginès P. (2017). Acute-on-chronic liver failure: An update. Gut.

[23] Gustot T. (2011). Multiple organ failure in sepsis: Prognosis and role of systemic inflammatory response. Curr. Opin. Crit. Care.

[24] Pettilä V., Hynninen M., Takkunen O., Kuusela P., Valtonen M. (2002). Predictive value of procalcitonin and interleukin 6 in critically ill patients with suspected sepsis. Intensiv. Care Med..

[25] Gonzalez-Casas R., Jones E.A., Moreno-Otero R. (2009). Spectrum of anemia associated with chronic liver disease. World J. Gastroenterol..

[26] Qamar A.A., Grace N.D., Groszmann R.J., Garcia–Tsao G., Bosch J., Burroughs A.K., Ripoll C., Maurer R., Planas R., Escorsell A. (2009). Incidence, prevalence, and clinical significance of abnormal hematologic indices in compensated cirrhosis. Clin. Gastroenterol. Hepatol..

